# The Cincinnati Medical News

**Published:** 1859-02

**Authors:** 


### THE CINCINNATI MEDICAL NEWS

Is the title of a monthly paper, edited and published by Prof. A. H. BAKER, M. D., of this city,—which should have been noticed in the November number, but was overlooked. This is a free, bold, and independent publication, and calculated to do much good in the profession, by exposing error and advocating truth. Generally there is too much disposition to "smooth things over." Give us the man who is always ready to "face the music," and such be believe Dr. Baker to be. We bespeak for the *Medical News* a large circulation. Price, fifty cents a year.

